# Integrating Clinical Presentation with Patient Encounter Experience and Community-Based Activities in the Pre-clerkship Curriculum. An Example of a Framework for Design, Implementation, and Evaluation

**DOI:** 10.1007/s40670-024-02235-1

**Published:** 2024-12-20

**Authors:** Asa Black, Richard Goodwin, Thomas I. Nathaniel

**Affiliations:** 1 School of Medicine Greenville, University of South Carolina, 701 Grove Rd, Greenville, SC 29605 USA; 2 College of Engineering and Computing, University of South Carolina, Columbia, SC, 29208 USA

**Keywords:** Patients, Family, Medical students, Integration

## Abstract

The urgent need to enhance medical students’ clinical problem-solving skills even in the pre-clerkship phase ushered in several initiatives in the medical school curriculum. One of such initiative is the introduction of clinical presentation activities combined with patient encounter experience and community-based activities in the first year of medical school. The study described the design, implementation, and evaluation of a medical school curriculum that integrates clinical presentation activities with patient encounter experience and a community-based educational program. For the clinical presentation, a two-tailed, Student’s *t*-test showed a significant and higher performance in the summative assessments when compared with the formative assessment (*P* < 0.01). The KR-20 value for the reliability was between 0.69 and 0.92. For the patient interaction experience and community-based stroke education program, medical students demonstrated a significant (*P* < 0.05) higher perception of their experience in all assessment categories in the post-test evaluation. An integrated curriculum that allows students to directly interact with patients, families, and community members may add a positive context and engagement experience to make a difference in the education of future physicians.

## Introduction

The need to advance both scientific and clinical knowledge for medical students in the pre-clerkship phase requires innovations in the medical school curricula. Various innovations and trends have been proposed [1, 2]. One focus is the need to enhance students’ clinical problem-solving skills during the pre-clerkship phase of the medical school curriculum. One such initiative is the introduction of clinical presentation [3] during the teaching of basic science to build concepts and contents that will help medical students better understand the pathophysiological processes linked to a given clinical condition. The rationale is that the reaction of the human body to an unlimited number of insults is restricted and enduring over time [4]. Therefore, it is very important to use a pedagogical strategy that helps medical students understand the process of progressing from symptoms to diagnosis of the clinical condition. One possibility is that if the teaching faculty members organize their teaching around these clinical presentations, then medical students will understand better all the common problems related to digestive or nervous system abnormalities. One of the expectations in the implementation of a clinical presentation is that for all clinical presentations, there should be a schema [5]. The schema is expected to stimulate critical thinking and reasoning processes in medical students, such that at each step of differentiation between the diagnostic categories, medical students should be able to use basic science knowledge, history, physical examinations, and related laboratory results [6]. Therefore, the clinical presentation is a scheme-driven search strategy and an inductive reasoning process [7]. In general, the primary goal of clinical presentation is to help students understand the process of progressing from symptoms to diagnosis of clinical problems.

While clinical presentation imparts medical students with theoretical and practical knowledge of disease processes with emphasis on education about diagnostic and treatment modalities, it does not provide the engagement skills that are very important for physicians to be able to deal with patients [8]. Therefore, not only are pedagogical strategies important, but methods that employ andragogy are paramount in this setting. We used clinical presentations to teach relevance — a principle central to teaching adult learners. Therefore, there is a need to link clinical presentation with patient encountering experiences such that medical students can interact with patients, their family members, and the community. This synergistic approach fosters awareness of the importance of human connection to improve students’ social interaction and communication skills. Therefore, incorporating early exposure of medical students in the pre-clerkship phase to patients into the medical school curriculum is a way to bring first-year students closer to patients during the pre-clinical coursework and before clinical rotations start in the third and fourth year of the medical school training [9]. The opportunity that patient-student interactions provide includes empathy [10] which cannot fully be taught through the analysis of clinical cases during a clinical presentation session or didactic teaching. Therefore, patients encounter experience for first-year medical students the raw human experience that unites both the doctor and the patient in the real world.

Community-based medical education programs have been reported to provide an adequate environment for medical students to learn more about health problems compared to hospital-based settings [11]. It allows the medical curriculum to directly contribute to addressing healthcare issues in the community. The quest to produce physicians who are technically and culturally competent and are willing to serve in the rural community requires that medical students be exposed to community-based activities. Most medical school curriculum do not combine clinical presentation with patient encounter experience with contextual learning of early exposure to community settings. This affects the ability of medical students in the first year to integrate the experience of seeing real-life contextual experience through students’ interaction with patients in the first year, combine this experience with their sessions in clinical presentation to help students understand the process of progressing from symptoms to diagnosis of clinical problems and understanding factors other than diseases that influence health issues in underserved communities [12]. This study addresses this gap by developing a more systematic medical curriculum that integrates clinical presentation, with patient encountering experiences and community-based activities in the first year of a medical neuroscience course. The goal is to help medical students develop strategies for future differential diagnosis of patients in a systematic manner by providing a synergistic but distinct one-on-one interaction with patients in the physician role to help develop empathy skills and a community-based education to promote socio-behavioral aspects of medical students in understanding factors affecting health problems in the primary healthcare setting.

## Method

The participants for this study were medical students in the first year of the medical school curriculum. A total of 52 medical students participated in this study. Fifty-two percent were female, and 48% were males. The average age was 23.0 ± 0.23, while the average Medical College Admission Test (MCAT) score was between 472 and 528, and the average GPA was 3.62.

### Description of the Medical Neuroscience Course

**Fig. 1:**
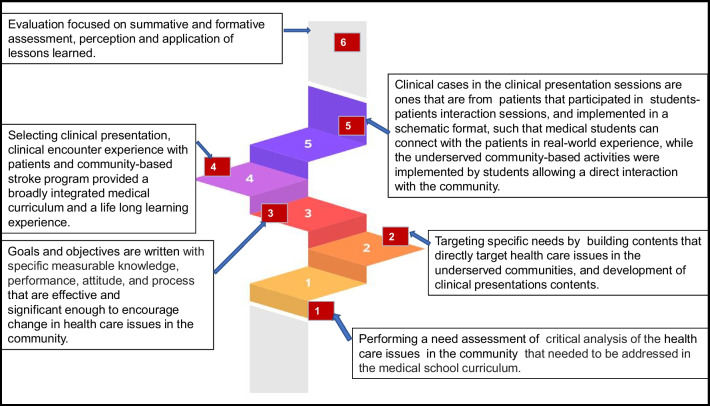
A modified six-step model approach for the development of an integrated curriculum that connects clinical presentation, students’ direct encounters with patients, and community-based stroke program

The idea of implementing clinical presentations for the medical neuroscience course was conceived during the course development by both basic and clinical faculty involved in the development of the curriculum. After a series of meetings, inputs from basic science and clinical faculty on how to develop clinical cases for each topic were agreed upon, including presentation methods, themes, case-based learning, and clinical presentations of the cases in a small group active learning session. Basic and clinical faculty worked together as a group to develop each of the cases and the number of cases for each topic. The Neuroscience course is mainly case-based clinical presentations in an integrated curriculum. Clinical presentations integrate biochemistry, cell biology, embryology, genetics, anatomy, histology, immunology, microbiology, nutrition, pathology, pharmacology, and physiology of the nervous system. The Integrated Practice of Medicine course runs concurrently with the Medical Neuroscience course to integrate doctoring skills each week to reinforce and enhance the information being taught in the course and the rest of the curriculum. Clinical presentation sessions were held every week in a small group session. The design of the clinical presentation session is presented in Fig. 1.

A total of 5 weeks comprised of 24 h per week was dedicated to the implementation of the neuroscience course. Clinical cases for each week were organized into different schemes to reflect the structure and functions of the nervous system. For example, week 1 focused on clinical cases of lesions of different blood vessels resulting in cortical and subcortical strokes or tumors that are frequently observed in local hospitals. Cases that cover structural and functional correlates of neuronal activity as the scheme was developed for the second week. These include cases of disruption of synaptic transmission and synaptic dysfunction in neurodegenerative and neurodevelopmental diseases. Cases for week 3 focused on lesions of vascular supply to other brain regions apart from cortical areas including the brainstem, pons, medulla, and spinal cord, while for week 4 most of the clinical cases were focused on the limbic, motor, and sensory systems, and special organs. Cases were developed for motor system abnormalities including Parkinson’s. Friday of week 4 (1:00–4:00 pm) was dedicated to students-patients encounters where students interacted with patients and their family members in a small group interactive session nonclinical session, while Tuesday of week 5 (8:00–3:00 pm) was dedicated to the community-based activities where medical students implemented a community-based education to contribute in addressing the healthcare issues in the community. This study was approved by IRB at the University of South Carolina School of Medicine Greenville in compliance with the ethical standards of the institutional committee.

### Description of the Design of the Integrated Clinical Presentation, Community-Based Activities, and Patient Encounter Experience

We used a modified Kern’s framework [13] for the design of all activities (see Fig. 1). For each step, we used educational principles and evidence. For example, for step one, we focused on specific community needs in healthcare issues. We interacted with people in the community to identify community healthcare issues that the curriculum can directly address. Through a focus group interaction with members of the community, scientific publications, and hospital data in the community, we identified specific healthcare issues including that the region is within the stroke belt area, and prominent risk factors for stroke including obesity, cardiovascular diseases, and diabetes among others are issues that contribute to the high prevalence of stroke and healthcare issues in the underserved community.

The second step focused on targeting health issues and building content. This informed our decision to develop clinical presentation content that directly targets healthcare issues in underserved communities. We were able to talk to individual patients and this informed our decision to include health issues prominent in the community in the contents of our integrated neuroscience clinical presentation cases. The third step focused on goals and objectives. Our educational objectives were set to be effective and significant enough to encourage change in healthcare issues in the community. For example, the integrated clinical presentation sessions were developed based on commonly encountered neurological presentations in the healthcare system in the community, and as identified in the need assessment outcome. Therefore, the goals and objectives were developed to allow us to be able to assess whether our medical students learned and internalized the expected outcomes. Most importantly, it allowed us to develop the main goals of the schema for each clinical presentation session including important information on clinical aspects such as history, physical examination, laboratory data, basic sciences, and health system science components to help students walk through the schema. Components of the health system included in each case were social determinants on individual patients, their families, and the community, health disparities, lifestyle medicine, high-value care, health policy and economics, etc.

In the fourth step, we focused on (1) how the educational strategies will engage the medical students as targeted learners involve and empower them to want to be influenced by the integrated clinical presentation session, (2) clinical encounter experience with patients that provides medical students the opportunities to directly interact with patients even in the pre-clerkship phase, and (3) a community-based stroke program that allows the curriculum to directly contribute to addressing stroke and related risk factors which was the main healthcare issue identified in the underserved community. The fourth step was the implementation phase (see full details below).

## Description of the Implementation Clinical Presentation Session

**Fig. 2:**
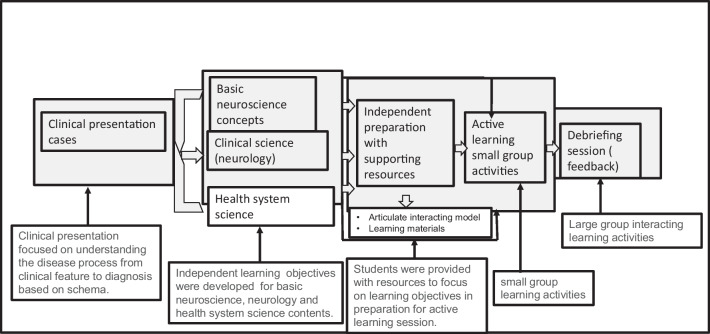
A summary of the design and implementation of the clinical presentation case-base session of the Neuroscience course

Most of the clinical cases for the clinical presentation session were cases discussed during the ward rounds, ward-based teachings, and outpatient clinics so that students can link the knowledge with the clinical practice. The cases were also the ones that were from patients who participated in student-patient interaction sessions (see “Description of Medical Students-Patients Encounter”), such that medical students can connect with the patients in real-world experience. The cases were developed by an interdisciplinary team that comprised clinicians and basic science faculty allowing integration. Each case comprised basic, clinical, and health system sciences objectives that were mapped through content integration, linking learning outcomes across basic, clinical, and system science contents. All cases were developed based on real patients and were accompanied by questions to stimulate a focused inquiry and self-directed learning. A total of 25 clinical presentations were developed and covered four schemes. Clinical presentation sessions were implemented on Fridays between 8:00 and 11:00 am. Didactic materials were taught between Mondays and Thursdays including laboratory activities. These materials prepared students for the clinical cases and were posted on the canvas platform a week before the implementation of the clinical presentation session. Students were able to assess the clinical cases 72 h before the session, giving them enough time to prepare for the clinical presentation session. The session was implemented using an integrated teaching strategy in a small group format. Medical students were divided into groups of 9–10 students per group for 5 groups. In the first 40 to 50 min, a general overview and schema was provided. The developed schema for all clinical presentations provided pathways that students can use for learning as well as problem-solving in clinical settings. Students were given a 10-min break and they reconvened to work with their groups to discuss and prepare for their responses to each of the questions that accompany each clinical case. Each case has an average of five questions per case for the five cases randomly distributed among the 9–10 groups and discussed in a time frame of 2 h. While each case was assigned to each group during the clinical presentation session, each student did not know which group or case his or her group would work on. Therefore, each student prepared for all five cases before the clinical presentation session. Each group had about 10–15 min for a case presentation followed by another 5–10 min for a questioning session by other groups to a particular group making presentations. This was followed by a 1-h debriefing session implemented by clinical and basic faculty in an integrated teaching format that allows multiple contributions by both basic and clinical faculty to provide a holistic learning of the materials. They provided a general summary and clarified any confusing concepts. The implementation of the clinical presentation session is summarized in Fig. 2.

### Description of Medical Students-Patients Encounter

Medical students were exposed to patients who were prototype neurological disorders and most of them were cases on whom clinical presentations occurred earlier (see “Description of the Implementation Clinical Presentation Session”). The selection of patients was based on neurological disorders and cases that clinical presentations occurred earlier. The cases are the ones discussed during the ward rounds and outpatient clinics. Students interacted with patients who presented with stroke, Alzheimer’s dementia, Parkinson’s disease, traumatic brain injury, cerebral palsy, epilepsy, primary lateral sclerosis, Charcot-Marie-tooth disorder, inclusion body myositis, multiple sclerosis, myasthenia gravis, and spina bifida. The purpose of the patient encounter session was to provide a real-world experience, where students can learn from the patients the humility and listening skills that make a good and compassionate clinician. Students were divided into 9–10 groups, and the session was held in a small group session in the medical school building to provide a nonclinical setting. This approach allowed an informal discussion where patients can share with medical students their diagnosis, their activities of daily living, their prognosis, and how the disorder affects their families. The session was implemented on the fourth week, on Friday between 1:00 pm and 4:00 pm. Each group was provided the opportunity to interact with a patient for about 30 min and alternated to meet with another patient for another 30 min. This implies that each student was able to meet with at least two different patients. During each session, they asked questions related to the neurological conditions. The patient’s family members who participated in the session responded to students’ questions and also shared their own experiences in working with the patients at home. After the session, medical students met in the lecture hall for the debriefing session. The group debriefing session provided the opportunity for medical students to share their interactive experiences and observations with others for another 2 h. All clinicians including neurologists, neurosurgeons, and neuroradiologists and basic science faculty including neuroscientists, biochemists, and pharmacologists participated in the session. This facilitates interprofessional/interdisciplinary teaching collaboration such that the team work together to achieve the common goal of training medical students by integrating basic science information with clinical concepts to advance fundamental understanding or solve clinical problems in a clinical presentation session.

The participating faculty further provided more discussion to further connect with clinical presentation sessions. Patients did not participate in the debriefing session. For each group, a representative provided information about the patient’s neurological condition, and demographic information, and then provided some reflections about what they learned from the interactions with the patient, how they were impacted emotionally, and lessons learned through experience and interactions with patients even in the pre-clerkship phase of an integrated medical curriculum.

### Development of Community-Based Activities

We develop a community-based stroke education program to contribute to addressing the identified healthcare issues and prominent risk factors for stroke including obesity, cardiovascular diseases, and diabetes that are prominent issues that contribute to the high prevalence of stroke. The community-based stroke program allows medical students to directly interact with members of the community and develop their cultural competency skills, while also educating them on healthy lifestyle activities to prevent stroke and reduce obesity and other risk factors of stroke. The program focused on middle school students in underserved communities. The focus on middle school students is because age (11–14 years) represents a significant developmental period when kids learn about the human body, the basic skills in using first aid, and how to respond during an emergency [7]. Therefore, providing a student-centered, hands-on learning educational program that guides mastering the scientific way of thinking and making decisions on how to provide basic help to anyone can enhance personal responsibility and their ability to adopt positive lifestyle choices to prevent stroke in the future [8]. In addition, middle school age is a good time to coach and equip them with knowledge about signs of stroke, such that if a parent or grandparent is a victim of stroke they can respond by calling 9–11.

**Fig. 3:**
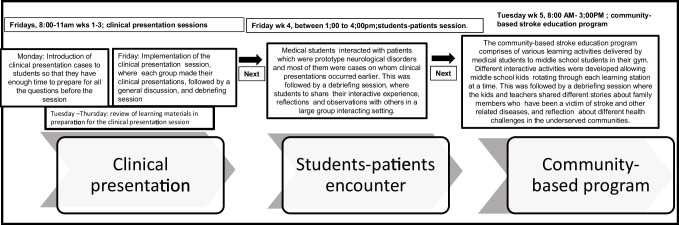
The sequence in the implementation of the integrated clinical presentation with patient encounter experience and community-based activities in the medical Neuroscience course

The program was implemented for a full duration of the school day, from 8:00 AM to 3:00 PM on Tuesday of the fifth week, and consisted of six periods that lasted 50 min each for the implementation of the program. The community-based stroke education program comprised various learning activities delivered by medical students to middle school students in their gym. First, a documentary on selected lifestyle habits and stroke was presented to middle school kids by medical students. Videos of healthy lifestyle activities associated with a lower risk of coronary heart disease, diabetes, and total cardiovascular disease and the impact of multiple lifestyle factors on the risk of stroke were presented. Medical students emphasized how a low-risk lifestyle that is associated with a reduced risk of multiple chronic diseases also may be beneficial in the prevention of stroke. Middle school kids also watched a video of younger stroke victims to demonstrate that while stroke can happen at any age, and is common among older people, teenagers may also be victims of stroke (Fig. 3).

Second, a total of five learning stations were created to implement the community-based stroke education program. Between 10 and 25 middle school students per station participated in different interactive activities rotating through each learning station at a time. The activities of each station were facilitated by medical students who provided guidance. For example, at station 1, middle school students learned the signs and symptoms of stroke with a large visual representation of each letter and activities corresponding to each letter. By associating each letter of the FAST signs that includes weakness of the face, arm, speech, and time to call 9–11 with a stroke symptom, middle school students learned the signs and symptoms of stroke interactively. The “Rethink Your Drink” represents the second station, where middle school students were educated by medical students about how reducing sugar intake could help prevent stroke. They were educated about the amounts of sugar that can be found in everyday drinks. At the third station, middle school students were educated about the salt content in different foods and how maintaining a healthy diet may help prevent stroke. Station 4 featured different activities, including racing, jumping, and pushups. Activities at station 4 emphasized the importance of exercise and physical activity in living a healthy lifestyle and preventing stroke. The last station featured seven habits of a healthy lifestyle and the seven habits of an unhealthy lifestyle. Middle school kids interacted with medical students and peers and discussed different healthy lifestyle activities that can help prevent stroke. The final phase was the debriefing session during which medical students interacted with middle school students, teachers, and others in underserved communities. The kids and teachers shared different stories about family members who have been a victim of stroke and other related diseases. This provided a profound reflection of learning for them during intensive exposure to community health problems. Through the program, they learned about health problems from family members and people who are victims different from the ones in the hospital-based settings. This allowed them to fully comprehend the essentials of primary healthcare issues at the beginning of their medical education, by reflecting on their experiences through the community-based stroke program.

### Evaluation

Evaluation of the clinical presentation was focused on the assessment of student’s performance in the formative and summative examinations. Students were provided with a weekly formative assessment to provide feedback for medical students’ learning. The format for the National Board of Medical Examiners (NBME) step 1 examination clinical vignette-style questions was used for the writing of both formative and summative questions. Although the formative assessments did not contribute to students’ final grades, the questions used in the formative assessments were very challenging to encourage critical thinking.

We focused on students’ perception of the impact of their encounters with patients and community-based programs in providing an integrated clinical presentation experience with contextual learning or more meaningful learning that can be enhanced by early exposure to community settings. Consequently, a descriptive cross-sectional survey was carried out to determine the level of perception in the patient encounter, using a protocol published by Alonso-Coello et al. [14]. The measurement instrument for the patient encounter was specifically designed to cover perceptions about (1) enhancing listening and communication skills, (2) insights and meaningful experience in medicine, and (3) social interaction, empathy, and clinical reasoning skills for a future career in the field of medicine. For the community-based stroke education program, the perception was focused on (1) interprofessional education and education measures, (2) social inequalities and determinants of healthcare, and (3) health issues in underserved communities. Seven statements were analyzed considering students’ perceptions about the encounter with patients and the community-based stroke education program. A 5-point Likert scale with a score of 1 = Strongly Disagree, 2 = Disagree, 3 = Neutral, 4 = Agree, and 5 = Strongly Agree was used to gather student feedback regarding the sessions. The questionnaire was completed online. The students did not receive any incentive for completing the questionnaire. EvaSys Survey Automation Suite version 7.1 was used to collect the data.

### Statistical Data Analysis

The mean percentage of students scoring ≥ 70% in each of the four themes that the summative questions were mapped to was computed. Percentage scores for formative assessment for the four themes were also computed. Cohen’s *d* value was determined to quantify the effect size between the summative and formative assessments, while values for KR-20 (Kuder–Richardson Formula 20 [15]) were used to compute the reliability of both the formative and summative examinations. This approach allowed us to determine the internal consistency and reliability of the performance [16]. KR-20 values ranged between 0.0 and 1.0; higher values indicate a more reliable test capability of discriminating between students with a higher performance who understand the test material versus those with low performance and a poorer understanding [17]. The results of the seven-item survey that assessed the perception of students were analyzed. Descriptive analyses were performed, and categorical data were presented in percentages. A two-tailed, Student’s *t*-test was used to determine student performance in the summative and formative assessments for each theme. The two-tailed test was also used to determine the pre- and post-evaluation of students’ perceptions for those that agree or strongly agree with the student’s patient encounter and community-based stroke education program.

## Results

The results for the formative and summative examinations administered including the reliability test along with a calculated Cohen’s *d* value to quantify the effect size between the summative and formative assessment are presented in Table 1. As shown in Table 1, all the formative and summative examinations had a KR20 value greater than 0.5, ranging between 0.69 and 0.92. The mean score achieved on the weekly formative examinations is broken down by each of the four themes comprising five cases for each theme. A two-tailed, Student’s *t*-test showed a significant and higher performance in the summative assessments when compared with the formative quizzes (*P* < 0.01). Table 2 presents the results of the seven-item survey that assessed medical students’ perception of interactive patient encounters during the medical neuroscience course. 89.3% of medical students either agreed or strongly agreed with the expectation that the students-patients interactive activity will improve their listening skills with patients, and provide valuable information regarding their ability to apply patient-centered communication. Students’ perception increased to 97.9% in the post-test evaluation and the increase was significant (*t* = 11.350, *P* < 0.05). Before the implementation of the program, the majority of the students (87.4%) expected that the session would provide increased insight into the patient’s quality of life and patient–physician relationships even in year 1 of their medical school career. Post-test evaluation indicated a 95.8% and a significant (*t* = 10.054, *P* < 0.05) increase in the perception. 85.4% of medical students agree or strongly agree with an expectation of a possible meaningful experience of the program connecting with clinical presentation sessions. The post-test evaluation revealed a 99.0% and significant (*t* = 12.640, *P* < 0.05) increase. 89.0% of medical students either agreed or strongly agreed with the expectation that they will learn lessons about empathy, and the diversity of patients’ experiences. This increased to 98.8% of medical students’ perception after the post-test evaluation (*t* = 9.065, *P* < 0.05). 89.8% of medical students expected the session to improve their clinical reasoning skills, including listening to the patient’s story or records, to help them improve their skills about what they think about and how they think in developing an action plan in the future as clinicians. The perception increased to 97.5% in the post-test evaluation (*t* = 13.086, *P* < 0.05). 88.1% of medical students expected the session to provide insight into how patients’ encounters with physicians, in the future trust in doctor-patient relationship. The perception increased to 96.3% and the increase was significant (*t* = 7.0645, *P* < 0.05). Overall, 93.5% of students either agreed or strongly agreed that the students’ interactive experience would meet or surpass their expectations in helping them with skills needed for effective engagement with patients and families, social interaction, and communication skills to enhance the learning experience of coping patients in the real world. The post-test evaluation revealed that the perception increased to 95.7%, but the difference was not significant (*t* = 0.014, *P* > 0.05).

**Table 1:** The results for the formative and summative examinations administered include the reliability test along with a calculated Cohen’s *d* value to quantify the effect size between the summative and formative assessment. The expectation was that 70% of students would score > 70 in the formative and summative assessments. As shown in the table, the target of 70% was met in all four themes. The effect size for this analysis (*d* = 2.01–3.41) was found to exceed Cohen’s convention for a large effect (*d* = 0.80), which reflected the magnitude of differences between the formative and summative assessments

Themes	Formative (% > 70 ± SD)	Summative (% > 70 ± SD)	*P*-value	Cohen’s *d* value	Formative (KR20)	*P*-value	Formative (KR20)
1.0 Structure and functions of the nervous system	69.00 (± 3.05)	90.0 (± 3.12)	< 0.01	2.01	0.69	< 0.01	0.79
2.0 Structural and functional correlates of neuronal activity	70.0 (± 3.21)	86.0 (± 2.97)	< 0.01	3.03	0.78	< 0.01	0.86
3.0 Vascular supply to brain regions	72.0 (± 4.01)	87.0 (± 4.01)	< 0.01	3.21	0.82	< 0.01	0.92
4.0 Sensory and motor systems	81.0 (± 3.56)	92.0 (± 3.28)	< 0.01	3.41	0.76	< 0.01	0.89

**Table 2:** The results of the seven-item survey that assessed medical students’ perception of interactive patient encounters during the Medical Neuroscience course. Descriptive analyses were performed, with categorical data presented in percentages for pre- and post-evaluation of students’ perceptions. Comparisons between pre- and post-intervention responses were analyzed using a two-tail Student’s *t*-test

	Pre-test (%)					Post-test(%)					*P*-value
	Strongly disagree	Disagree	Neutral	Agree	Strongly agree	Strongly disagree	Disagree	Neutral	Agree	Strongly agree	
1.0 The session improves my listening skills with patients and provided valuable information regarding ability to apply patient-centered communication in future practice	0	0	10.7	47.3	42.0	0	0	2.1	20.4	77.5	< 0.05
2.0 The session will provide an increased insight into the patients’ quality of life and patient–physician relationships	0	0	12.6	43.2	44.2	0	0	4.2	17.8	78	< 0.05
3.0 The session provides meaningful experience in meeting patients with problems or diseases in alignment with the clinical presentation sessions	0	0	14.6	36.7	48.7	0	0	1.0	23.7	75.3	< 0.05
4.0 The session will help me to learn lessons about empathy, and the diversity of patients’ experiences	0	0	11.0	43.0	46.0	0	0	1.2	12.9	85.9	< 0.05
5.0 The opportunity of patient interactions, will help develop clinical reasoning skills about what they think about and how they think in developing an action plan in the future as clinicians	0	0	10.2	20.4	69.4	0	0	2.5	14.7	82.8	< 0.05
6.0 This session provides insight on how patients’ encounters with physician, in future doctor-patient relationship	0	0	11.9	39.1	49.0	0	0	3.7	11.2	85.1	< 0.05
7.0 Meeting the patients will help with the skills needed for effective engagement with patients and families, social interaction and communication skills to enhance learning of coping with real world patients	0	0	6.5	45.1	48.4	0	0	4.3	21.1	74.6	< 0.05

Table 3 presents the results of the seven-item survey that assessed medical students’ perception of community-based stroke education programs. 86.6% of medical students expected that the program would provide an opportunity for interprofessional education by working with different groups. The perception increased to 100% among those who agree or strongly agree (*t* = 18.085, *P* < 0.05) that the program has improved their readiness for interprofessional community-based learning. Before the intervention, 87.0% expected that the session would adopt adequate education measures for prevention actions for the target population to achieve behavioral and health change. The perception improved significantly to 100% (*t* = 17.045, *P* < 0.05) for students who agreed or strongly.

**Table 3:** The results of the seven-item survey that assessed medical students’ perception of community-based stroke education programs implemented in the underserved community for middle school students. Descriptive analyses were performed, with categorical data presented in percentages for pre- and post-evaluation of students’ perception. Comparisons between pre- and post-intervention responses were analyzed using a two-tail Student’s *t*-test

	Pre-test					Post-test					*P*-value
	Strongly disagree	Disagree	Neutral	Agree	Strongly agree	Strongly disagree	Disagree	Neutral	Agree	Strongly agree	
1.0 The program provides an opportunity for interprofessional education by working with different groups	0	0	0	42.0	58.0	0	0	0	18.0	82.0	< 0.05
2.0 The session adopts adequate education measures for prevention actions and determinants of health	0	0	1.2	50.8	52.0	0	0	0	21.0	79.0	< 0.05
3.0 The program helped me to gain a better understanding of social inequalities in health care issues	0	0	2.1	49.4	48.5	0	0	0	13.0	87.0	< 0.05
4.0 The program helped to understand health determinants, and health literacy, and empathy for people affected by these issues	0	0	0	54.0	46.0	0	0	0	14.7	85.3	< 0.05
5.0 Participating in the community program for healthy life style is empowering to me and related to contents of our curriculum	0	0	3.1	45.0	51.9	0	0	0	17.0	83.0	< 0.05
6.0 The program provided the opportunity to gain experience in health education, prevention, and development of skills in oral communication, and group work	0	0	3.2	47.5	49.3	0	0	0	17.9	82.1	< 0.05
7.0 Provided an opportunity to better understand public health issues in the underserved community	0	0	0	47.0	53,0	0	0	0	16.0	84.0	< 0.05

87.2% of students expected that the program would help them gain a better understanding of socio-economic factors in the underserved community that contribute to social inequalities in healthcare issues. After the program, 100% of the students agreed or strongly agreed (*t* = 17.076, *P* < 0.05) that the program provided a better understanding of socio-economic factors in the underserved community. 85.3% of medical students expected that the community-based stroke education program would help them understand social determinants of health, and provide community-based health literacy interventions to improve the health of the population. Post-test evaluation indicated that 100% of medical students agree or strongly agree (*t* = 19.063, *P* < 0.05). 89.5% expected the program to improve primary medical education, by enhancing undergraduate medical education with the primary healthcare context. The perception increased to 100% (*t* = 16.072, *P* < 0.05) after the implementation of the program. 88.3% of medical students expected the program to provide the opportunity to gain experience in health education and prevention and raise awareness about major health issues in the community. Following the intervention, the perception increased to 100% (*t* = 12.078, *P* < 0.05). 87.4% expected the program to provide an opportunity to better understand public health issues in the underserved community. The perception significantly (*t* = 16.075, *P* < 0.05) increased to 100% in the post-test evaluation.

## Discussion

### Clinical Presentation Session

The current study investigated the design, implementation, and evaluation of clinical presentation activities combined with patient encounter experience and community-based stroke education program in the first year of medical school. Students’ performances were higher in the summative when compared with the formative assessments. This is because the questions used in the formative assessments were more challenging and the goal was to encourage deeper levels of learning to reflect the format and difficulty anticipated for the final summative assessment. Therefore, medical students developed their critical thinking skills in clinical issues through clinical presentation and in the formative assessments, which were more challenging written clinical vignette questions than the summative examination. They were able to construct clinical outcomes in a logical and systematic sequence that may be applicable in real-life clinical practice settings. Following repeated practice in the solving of clinical problems through analysis of clinical cases in the clinical presentations, they developed a pattern recognition skill in patient problems. Therefore, the schematic structural framework provided in our clinical presentation and formative questions provided the background and direction that helped medical students, especially in the pre-clerkship phase to shape their clinical reasoning by organizing their knowledge in ways that can help solve specific clinical problems [18]. The strong performance in the summative assessment indicated that they were able to retain and use basic science concepts to provide a contextual analysis of specific clinical problems [19], allowing effectiveness in chronological and logical thinking resulting in a strong performance in summative assessment.

### Patients-Interaction Encounter Session

Following the implementation of clinical presentations, we then exposed medical students to directly interact with patients with neurological disorders and most of them were actual cases on whom clinical presentation learning activities occurred earlier. Medical students interacted with stroke, Alzheimer’s dementia, Parkinson’s disease, traumatic brain injury, cerebral palsy, epilepsy, primary lateral sclerosis, Charcot-Marie-tooth disorder, inclusion body myositis, multiple sclerosis, myasthenia gravis, and spina bifida patients in the patients encounter session. In addition, the patient’s encounter experience was based on neurological presentations in the healthcare system as identified in the need assessment outcome. We provided the opportunity for medical students to directly connect with the patients in real-world experience in a nonclinical setting.

In the post-test evaluation of the patient encounter with medical students, we observed that medical students demonstrated a higher perception of their experience in all assessment categories. Patients and family members shared their personal experiences as well as the treatment outcomes, family challenges, and the importance of a physician who is honest, empathetic, and open with his or her patients. Medical students learned from patients about the humility, listening, and communication skills that make a clinician a good doctor. This is important as effective provider communication skills have been reported to be a key factor in ensuring better patient outcomes, reduced medical errors, and greater compliance with treatment plans [20, 21]. The patient encounter experience provided an opportunity for students to hear directly from patients about their experiences and standpoints on their health conditions [22]. Communication has also been reported to help in strengthening the conceptualization of the patient perspective by identifying aspects that, from doctors’ point of view, are important to address during a consultation to build a partnership with patients in receiving better healthcare [23, 24]. In general, our finding also reveals that patient interactions in an integrated medical curriculum during the first year has a significant impact on laying the foundation for their future clinical practice by fostering essential communication skills, building empathy, and providing real-world context to theoretical knowledge, while later interactions continue to improve these abilities and allow for more complex patient care management. Therefore, early exposure to patients is critical for building confidence and shaping a patient-centered strategy to medicine, allowing medical students to better understand the social and psychological context associated with a patient’s health condition, and this can inform future treatment decisions.

While clinical presentation activities may enhance medical students theoretical and pragmatic knowledge of disease processes with emphasis on diagnostic and treatment modalities [25, 26], they may not address the engagement skills that are very important in interacting with patients. We address this issue by providing the opportunity for medical students to interact with patients and their families in the patient encounter experience. A major rationale for patient encounters is that the trust and lessons that emerge between both parties when patients tell their stories cannot be entirely communicated through lessons in the clinical presentations. Our findings indicate that medical students perceived the encounter with patients to have provided them with meaningful experience, they learned lessons about empathy, and the diversity of patients’ experiences, clinical reasoning skills, future trust in the doctor-patient relationship, and skills needed for effective engagement with patients and families in their future practice. This is because patients shared with medical students about how much the quality of their care is connected to the physician’s empathy, which improves patient satisfaction [22], treatment compliance [27, 28], and clinical outcomes [28], as most patients indicate that they were more likely to follow their treatment plan and practice self-care when they feel heard and understood by their physicians. Therefore, our approach provided multiple benefits including changes in students’ attitudes toward patients, knowledge, and understanding related to patient-doctor communication, as well as new insights into psychosocial aspects of patients’ everyday life with the disease. Finally, by including patients and families in the curriculum, we created strong partnerships in the development of medical education programs and culture where partnerships with patients, families, and communities added a positive context to make a difference in the education of future doctors.

### Community-Based Activities

Our need assessment identified specific healthcare issues including that the region is within the stroke belt area, and obesity and cardiovascular diseases, including diabetes, are prominent healthcare issues in the community. This informed our decision to develop community-based stroke education that allowed the curriculum to directly contribute to addressing healthcare issues in the community including stroke and related risk factors. We observed that medical students demonstrated a higher perception of the program for their improved interprofessional community-based learning, adequate education measures in the program for prevention actions and behavioral and health change, and a better understanding of socio-economic factors in the underserved community, social inequalities, and determinants of health. Therefore, the community-based stroke education program directly targeted healthcare issues implemented by medical students who are committed to serving rural communities. It provided the opportunity for the curriculum to contribute to providing a practical solution to community needs.

Medical students worked in groups that comprised nurses, clinicians, and biomedical faculty members to implement the community-based stroke education program. Therefore, our program provided contextual learning with other health professions that introduced the concept of interprofessional collaborative practice [29–31] with a model of underserved community-based care. Medical students agreed or strongly agreed that the community-based stroke education program adopted adequate education measures for prevention actions that targeted underserved populations to achieve behavioral and health change. They strongly agreed that the program contributed to a better understanding of social inequality in healthcare issues, and social determinants of health. Our finding is supported by other studies [32–34] that community-based education may promote socio-behavioral aspects of medical students in understanding other factors than diseases that influence clinical conditions. Such factors include the social determinants of health, the conditions in which people are born, grow, live, work, and age affected by health issues [35, 36]. Therefore, our community-based education program allowed medical students to fully understand the influence of social determinants on individual patients, their families, and the community, and they reflected on learning during intensive exposure to community health problems [37].

Community-based educational activities are reported to provide a conducive environment to educate medical students more about health problems when compared to hospital-based settings [11]. In our community-based stroke education program, medical students learned the context of the epidemiological change, where a paradigm shift from disease-oriented care toward disease prevention was a major focus [38]. Therefore, we integrated community-based medical education in the delivery of integrated medical education in a specific social context, where medical students were part of the communities and actively participated in the implementation of the program to reduce the impact of stroke and related risk factors in the underserved community.

### Limitations

This study has some limitations. This was a pilot study at a single institution for a single course, and the results may not be generalizable to other institutions. However, integrating patient interactions in a clinical presentation curriculum of an integrated curriculum and allowing medical students to participate in community-based activities can be adapted for use in other institutions. While we collected data on students’ perceptions, we should have collected data on teachers as well as community members. Teaching foundational science in the clinical environment is challenging, requiring supervising clinicians to have the time and the knowledge to participate during patient encounter sessions. The long-term outcomes of this curriculum innovation are unknown/remain to be seen. Furthermore, there are opportunities for us to provide physician faculty development to help them teach foundational science at the depth that is expected in the clinical presentation sessions. Such a faculty development program should entail implementing activities to improve skills in designing, developing the objectives that integrates basic, clinical, and health system science and the implementation of a clinical presentation session. It should also include building active learning strategies to help students in solving the clinical problems. Such activities can enhance support curriculum innovation by helping faculty members develop the skills and knowledge they need to teach and facilitate student learning.

## Conclusion

In this study, we designed and implemented an integrated clinical presentation with community-based activities and patient encounter experience in a clinical neuroscience course of a medical school curriculum. The contents did not only focus on the disease process in the clinical presentation component of the curriculum but also focused on the distinctive relation between physicians and patients. Engaging medical students in community-based education provided training in the essentials of primary healthcare from the first year of their medical education, such that they can be future agents of change. In addition, medical students gained an insight into what it meant to be a patient who received the diagnosis and how lives and entire families are impacted by different neurological conditions, including the importance of engaging patients and their families in healthcare.
